# Psychosocial and economic impact of rheumatic diseases on caregivers of Mexican children

**DOI:** 10.1186/s12969-021-00524-2

**Published:** 2021-03-17

**Authors:** Brenda de Jesús Fortuna-Reyna, Ingris Peláez-Ballestas, Fernando García-Rodríguez, Enrique Faugier-Fuentes, Samara Mendieta-Zerón, Ana Victoria Villarreal-Treviño, Sara Georgina Rosiles-De la Garza, Greta Reyes-Cordero, Sol Jiménez-Hernández, Jessica Haydee Guadarrama-Orozco, Manuel Enrique de la O-Cavazos, Nadina Rubio-Pérez

**Affiliations:** 1 Department of Pediatrics, Universidad Autónoma de Nuevo León, Hospital Universitario “Dr. José E. González”, Madero y Gonzalitos SN, Col. Mitras Centro, C.P, 64460 Monterrey, Mexico; 2 Rheumatology Unit, Hospital General de México “Dr. Eduardo Liceaga”, Mexico City, Mexico; 3 Hospital Infantil de México Federico Gómez, Servicio de Reumatología, Mexico City, Mexico; 4 Instituto de Seguridad Social del Estado de México y Municipios, Hospital Regional Toluca, Toluca, Mexico; 5 Hospital Infantil de Especialidades del Estado de Chihuahua, Facultad de Medicina y Ciencias Biomédicas, Universidad Autónoma de Chihuahua, Chihuahua, Mexico; 6 Departamento de Cuidados Paliativos y Calidad de Vida, Hospital Infantil de México Federico Gómez, Mexico City, Mexico

**Keywords:** Pediatric rheumatic diseases, Caregiver, Family, Impact, Questionnaire

## Abstract

**Background:**

Pediatric rheumatic disease (PRD) patients and their caregivers face a number of challenges, including the consequences of the PRD in patients and the impact on multiple dimensions of the caregivers’ daily lives. The objective of this study is to measure the economic, psychological and social impact that PRD has on the caregivers of Mexican children.

**Methods:**

This is a multicenter, cross-sectional study including primary caregivers of children and adolescents with PRD (JIA, JDM and JSLE) during April and November, 2019. A trained interviewer conducted the CAREGIVERS questionnaire, a specific, 28-item multidimensional tool validated to measure the impact on different dimensions of the lives of caregivers. Sociodemographic, clinical, and healthcare system data were collected for further analysis.

**Results:**

Two hundred participants were recruited (women 169, 84.5%, aged 38 [IQR 33–44] years); 109 (54.5%) cared for patients with JIA, 28 (14%) JDM and 63 (31.5%) JSLE. The healthcare system was found to be determinant on the impact of the disease. The emotional impact was higher in all the participants, regardless of the specific diagnoses. The social dimension showed significant differences regarding PRD, healthcare system, time to reach the center, presence of disability, active disease, cutaneous and systemic manifestations, treatment and partner. Financial and work impacts were more frequent in those caring for JSLE and less so in those with a partner. Family relationships changed in 81 caregivers (25 [12.5%] worsened and 56 [28%] improved). No variables affecting spirituality were found. For caregivers without a partner, the social networks impact increased.

**Conclusion:**

The influence of sociodemographic factors can be devastating on families with children with a PRD. These data will help physicians to identify the areas with the greatest need for intervention to achieve comprehensive care for caregivers and their patients.

**Supplementary Information:**

The online version contains supplementary material available at 10.1186/s12969-021-00524-2.

## Background

Pediatric rheumatic diseases (PRD) are a heterogeneous group of disorders characterized by inflammation of connective tissue, especially the joints, blood vessels and skin [1]. The incidence varies by region and disease, between 2 and 13.9 per 100,000 children annually for Juvenile Idiopathic Arthritis (JIA) [2–8], 0.18 and 0.32 for Juvenile Dermatomyositis (JDM) [9–11] and 0.36 to 2.5 for Juvenile Systemic Lupus Erythematosus (JSLE) [12]. In Mexico, information on the epidemiology of PRD is scarce and vague [13–15].

PRD patients and their caregivers face a number of challenges. These include the consequences of the PRD in patients and the impact on multiple dimensions of the caregivers’ daily lives. Caregivers deal with the patients’ unlikelihood of carrying out activities appropriate to their age, restricted mobility, problems derived from school absenteeism, fatigue, pain and adverse events of medication [16]. Furthermore, patient hospitalizations, treatments and sequelae also have an impact on caregivers [17, 18]. It has been shown that the most affected dimensions in caregivers of patients with JSLE were related to their physical, mental, emotional and social functioning, in addition to the general perception of the health of their children [19]. Moreover, members of the affected family experienced feelings of grief, helplessness, aggressiveness, guilt, ambivalence, injustice or fear of the future and may suffer psychological, physical collapse or abandonment of the patient [17].

After a literature review focused on the impacts of PRD on caregivers, it was identified that there was no multidimensional instrument that would measure the impact that PRD had on the daily lives of caregivers of children and adolescents with these diseases. For this reason, our group developed and validated the “Impact of Pediatric Rheumatic Diseases on Caregivers Multi-assessment Questionnaire” (CAREGIVERS questionnaire) to measure the impact on caregivers of children with JIA with the objective of creating risk profiles and perform specific interventions to lessen the impacts on caregivers [20]. With a validated instrument, measuring the impact on caregivers was necessary to understand the impact better and be able to improve the well-being of caregivers and that of their patients. Additionally, we rationalize that the value of the questionnaire could be applied to other PRD with similar features and potential disabilities, such as JSLE and JDM.

The objective of this study is to measure the economic, psychological and social impacts that PRD have on the caregivers of Mexican children and the factors associated with these impacts.

## Patients and methods

### Study design

This is a cross-sectional study in which primary caregivers of children and adolescents with PRD were prospectively and consecutively included between April and November, 2019.

### Participants

The participants for this study were primary caregivers of both genders, over 18 years of age, of patients with a confirmed diagnosis of JIA (according to the criteria of the International League of Associations for Rheumatology) [21], JSLE (according to classification criteria of the American College of Rheumatology) [22] and JDM (fulfilling classification criteria of Bohan and Peter) [23, 24] that were being treated in the Pediatric Rheumatology Services of four public hospitals and in private consultation with the researchers.

Participants had to be close family members (parents, older siblings, grandparents, aunts or uncles) that served as the primary support, lived with the patient and were not professional caregivers.

Potential participants were excluded for having patients hospitalized in the four weeks prior to participating in the study, for being diagnosed with a chronic disease themselves, for having more than one patient in their care and for refusing to participate.

### Study procedures

During the routine follow-up appointment and after explaining the study and obtaining the participant’s informed consent, a trained interviewer invited the participant to a private room and conducted the CAREGIVERS questionnaire. The CAREGIVERS questionnaire is a tool that was designed and validated to measure the impact on different dimensions of the life of the caregiver of a patient with JIA. The tool includes 28 items that assess the emotional, social, family, economic and labor impacts, in addition to the caregiver-patient relationship, partner relationship, spirituality (religion or personal beliefs) and social networks. The CAREGIVERS questionnaire has an application time of approximately 15 min [20]. The time to complete each questionnaire was reported during the duration of the study.

In addition to the CAREGIVERS questionnaire, a sociodemographic data collection sheet was developed specifically for the study and includes data of the participants and of the patients’ diseases. The sociodemographic data were obtained through the interviews with the participants and/or review of the clinical records of the patients. Data included age, gender, occupation, level of education, marital status, time it takes to reach the treatment center, diagnosis, place of residence, hospital where the patient is cared for, main clinical manifestations, treatment being used for the disease, previous hospitalizations, presence of disability and activity of the disease evaluated by the physician. Also, the use of any treatment was considered if it was reported by the parent and/or was in the clinical record, regardless of the administration, dose or duration. The research team defined “low level of education” as completing a formal education process for nine years or less.

The patient’s health care system was defined as partial coverage (PartC) when the center covered medical appointments and hospitalizations (subsidized by the state), but not medications and other supplies. Full coverage (FullC) was when the center covered ambulatory, hospital care, medications, physiotherapy, devices, and even caregiver disability. Private coverage (PRI) when the expenses related to the care were fully covered by the patient and the patient’s family, including medical expenses insurance.

### Missing data and imputation

This study had no data imputation. Instead, the number of cases included in each calculation is specified. The tables show the absolute frequency over the total number of cases that report the variable and the calculation of the corresponding percentage.

### Statistical analysis

The study used descriptive statistics with frequencies and measures of central tendency and dispersion of the sociodemographic characteristics of the participants and the patients’ clinics. A univariate analysis was performed with the interview responses of the CAREGIVERS questionnaire and the sociodemographic, clinical and health system variables using the Chi square, Mann-Whitney U and Kruskal-Wallis tests. Statistical significance was considered when *p <* 0.05.

During the analysis, the clinical manifestations of the patients were grouped into cutaneous (any cutaneous feature reported by the caregiver or clinical record), musculoskeletal (arthritis and myositis) and systemic (including neurological, hematological, pulmonary, gastrointestinal, cardiovascular, nephritis manifestations, serositis, adenomegaly, hepatomegaly, hepatitis, thyroiditis and uveitis). The treatments were divided into four groups: 1) non-steroidal anti-inflammatory drugs (NSAID), 2) disease modifying drugs and synthetic immunosuppressants (DMARD), 3) systemic glucocorticoids (GC) and 4) biological immunosuppressive therapies (bDMARD).

To simplify the analysis, a scoring system was established for each of the items on the CAREGIVERS questionnaire. Items were excluded if considered contextual in Emotional Impact (“What concerns you the most about your child’s/patient’s rheumatic disease?”), and on Social Networks (“Have you searched for information about your child’s/patient’s rheumatic disease on the internet?” and “Have you used social media to communicate with other parents/caregivers of children who have the same rheumatic disease as your child/patient?”). Among the researchers of this study, the scoring was made by consensus, basing the discussion on a clinical context; the scores were based on a summatory of the items by dimension in ascending order. The higher the score, the greater the impact (Supplementary Table 1).

All statistical analysis conducted for the study was done using the Stata V.16 statistical program.

### Ethical considerations

This research received approval by the Ethics Committee of the main study center (“Dr. José E. González” University Hospital with registration code PE19–009) and subsequently, each participating center obtained approval by its local committee. Written informed consent was collected from each participant.

The confidentiality of the participants was maintained at all times by completing the questionnaires and registering the participants anonymously.

## Results

### Demographics and participants profile

Two hundred participants were recruited, most of whom were women (169, 84.5%) with a median age of 38 (IQR 33–44) years; 109 (54.5%) cared for patients with JIA, 28 (14%) for patients with JDM and 63 (31.5%) for patients with JSLE.

Most of them went to hospitals with PartC (147, 73.5%), required more than one hour to reach the center (131/168, 78%), had low educational levels (89/166, 53.6%), were housewives (90/168, 53.6%) and had a relationship with a partner (123/167, 73.7%). Table 1 shows the comparison of the sociodemographic variables among the PRD. The participants geographical representation was of 16 states (out of 32 in the country), while the majority came from three states. There were no participants from the northwestern and southeastern regions of the country (Supplementary Fig. 1).

**Table 1:** Comparison of demographic data of caregivers by PRD

		Total 200 n (%)	JIA 109 n (%)	JDM 28 n (%)	JSLE 63 n (%)	*p value*^a^
Age in years, median (IQR)		38 (33–44)	37.5 (32–42)	36.5 (33–43.5)	40 (36–46)	0.01^b^
Female		169 (84.5)	95 (87.2)	21 (75)	53 (84.1)	0.09
Healthcare system	PartC	147 (73.5)	74 (67.9)	24 (85.7)	49 (77.8)	0.14
	FullC	43 (21.5)	28 (25.7)	2 (7.1)	13 (20.6)	
	PRI	10 (5)	7 (6.4)	2 (7.1)	1 (1.6)	
Occupation	Housewife	90/168 (53.6)	50/87 (57.5)	16/26 (61.5)	24/55 (43.6)	0.19
	Paid employment	78/168 (46.4)	37/87 (42.5)	10/26 (38.5)	31/55 (56.4)	
More than one hour to the center		131/168 (78)	60/87 (69)	22/26 (84.6)	49/55 (89.1)	0.01
With partner		123/167 (73.7)	69/86 (80.2)	21/26 (80.8)	33/55 (60)	0.02
Educational level	≤ 9 years	89/166 (53.6)	48/87 (55.2)	16/25 (64)	25/54 (46.3)	0.31
	> 9 years	77/166 (46.4)	39/87 (44.8)	9/25 (36)	29/54 (53.7)	

*PRD* Pediatric Rheumatic Disease, *IQR* Interquartile range, *JIA* Juvenile Idiopathic Arthritis, *JSLE* Juvenile Systemic Lupus Erythematosus, *JDM* Juvenile Dermatomyositis, *PartC* Partial Coverage, *FullC* Total Coverage, *PRI* Private

^a^ Analyzed with Chi square test

^b^ Analyzed with Kruskal-Wallis test

### Clinical data of the patients

The patients had a median age of 13 (IQR 10–15) years, most of them female (134, 67%). The main symptoms during the course of PRD were musculoskeletal (161/193, 83.4%). The most prevalent clinical manifestations were articular in JIA (109, 100%, polyarticular 66, 60.6%); cutaneous (28, 100%) and myositis (27, 96%) in JDM; and cutaneous (39/56, 69.6%), hematological (29/56, 51.8%) and renal (17/56, 30.4%) in JSLE.

DMARD were the most used treatments (168/179, 93.9%); 97/174 presented a hospitalization history (55.7%), 43/174 some disability (24.7%) and 87/174 an active disease at the time of data collection (50%). The details of the comparisons between groups by PRD are shown in Table 2.

**Table 2:** Comparison of demographic and clinical data of patients by PRD

		Total 200 n (%)	JIA 109 n (%)	JDM 28 n (%)	JSLE 63 n (%)	P value*
Age in years, median (IQR)		13 (10–15)	12 (9–15)	11 (8.2–13.7)	14.5 (12–16)	0.01**
Female		134 (67)	66 (60.6)	19 (67.9)	49 (77.8)	0.14
JIA category	ERA	NA	6 (5.5)	NA	NA	NA
	Oligoarticular		8 (7.3)			
	Polyarticular		66 (60.6)			
	Psoriatic		1 (0.9)			
	Systemic		16 (14.7)			
	Undifferentiated		12 (11)			
Cutaneous symptoms		82/193 (42.5)	15 (13.8)	28 (100)	39/56 (69.6)	< 0.001
Musculoskeletal symptoms ^a^		161/193 (83.4)	109 (100)	27 (96.4)	25/56 (44.6)	< 0.001
Systemic symptoms ^b^		62/193 (32.1)	11 (10.1)	4 (14.3)	47/56 (83.9)	< 0.001
Treatment	NSAID	80/179 (44.7)	61/97 (62.9)	5/27 (18.5)	14/55 (25.5)	< 0.001
	DMARD	168/179 (93.9)	90/97 (92.8)	26/27 (96.3)	52/55 (94.5)	0.77
	GC	82/179 (45.8)	18/97 (18.6)	25/27 (92.6)	39/55 (70.9)	< 0.001
	bDMARD	53/179 (29.6)	49/97 (50.5)	1/27 (3.7)	3/55 (5.5)	< 0.001
Hospitalization		97/174 (55.7)	29/93 (31.2)	23/26 (88.5)	45/55 (81.8)	< 0.001
Disability		43/174 (24.7)	19/93 (20.4)	16/26 (61.5)	8/55 (14.5)	< 0.001
Active Disease		87/174 (50)	34/93 (36.6)	20/26 (76.9)	33/55 (60)	< 0.001

*PRD* Pediatric Rheumatic Disease, *IQR* Interquartile range, *NA* Does not apply, *ERA* Enthesitis related to Arthritis, *NSAID* Nonsteroidal anti-inflammatory drugs, *DMARD* Disease-modifying antirheumatic drugs, *GC* Systemic Glucocorticoids, *bDMARD* Biological therapy, *JIA* Juvenile Idiopathic Arthritis, *JSLE* Juvenile Systemic Lupus Erythematosus, *JDM* Juvenile Dermatomyositis

^a^: Includes arthritis and myositis

^b^: Includes neurological, hematological, pulmonary, gastrointestinal, cardiovascular, nephritis, serositis, adenomegaly, hepatomegaly, hepatitis, thyroiditis and uveitis manifestations

* Analyzed with Chi square test

** Analyzed with Kruskal-Wallis test

### Differences by health system

No differences were found in gender distribution or partner relationship of caregivers between health-care systems. However, those in PRI had a higher proportion of paid work, required less than one hour to reach the center and had a higher education level when compared to PartC and FullC (Table 3).

**Table 3:** Comparison of demographic data of caregivers by healthcare system

		Total 200 n (%)	PartC 147 n (%)	FullC 43 n (%)	PRI 10 n (%)	*p value*^a^
Age in years, median (IQR)		38 (33–44)	37 (32–44)	41.5 (36.5–44)	37.5 (33.5–48)	0.03^b^
Female		169 (84.5)	125 (85)	37 (86)	7 (70)	0.30
Occupation	Housewife	90/168 (53.6)	72/120 (60)	17/42 (40.5)	1/6 (16.7)	0.02
	Paid employment	78/168 (46.4)	48/120 (40)	25/42 (59.5)	5/6 (83.3)	
More than one hour to the center		131/168 (78)	104/120 (86.7)	27/42 (64.3)	0/6 (0)	< 0.001
With partner		123/167 (73.7)	84/120 (70)	35/41 (85.4)	4/6 (66.7)	0.14
Educational level	≤ 9 years	89/166 (53.6)	75/119 (63)	14/41 (34.1)	0/6 (0)	< 0.001
	> 9 years	77/166 (46.4)	44/119 (37)	27/41 (65.9)	6/6 (100)	

*IQR* Interquartile range, *PartC* Partial Coverage, *FullC* Total Coverage, *PRI* Private System

^a^ Analyzed with Chi square test

^b^ Analyzed with Kruskal-Wallis test

The patients presented differences in age, prevalence of cutaneous manifestations, treatments and outcomes when comparing health-care systems (Table 4).

**Table 4:** Comparison of demographic and clinical data of patients by healthcare system

		Total 200 n (%)	PartC 147 n (%)	FullC 43 n (%)	PRI 10 n (%)	P value*
Age in years, median (IQR)		13 (10–15)	12 (9–15)	14 (12–16)	13.5 (10.7–15.2)	0.01**
Female		134 (67)	95 (64.6)	32 (74.4)	7 (70)	0.85
Cutaneous symptoms		82/193 (42.5)	71/140 (50.7)	8 (18.6)	3 (30)	0.01
Musculoskeletal symptoms ^a^		161/193 (83.4)	120/140 (85.7)	32 (74.4)	9 (90)	0.19
Systemic symptoms ^b^		62/193 (32.1)	48/140 (34.3)	11 (25.6)	3 (30)	0.56
Treatment	NSAID	80/179 (44.7)	70/126 (55.6)	3 (7)	7 (70)	< 0.001
	DMARD	168/179 (93.9)	122/126 (96.8)	36 (83.7)	10 (100)	0.01
	Glucocorticoid	82/179 (45.8)	72/126 (57.1)	7 (16.3)	3 (30)	< 0.001
	bDMARD	53/179 (29.6)	24/126 (19)	24 (55.8)	5 (50)	< 0.001
Hospitalization		97/174 (55.7)	77/121 (63.6)	19 (44.2)	1 (10)	0.01
Disability		43/174 (24.7)	41/121 (33.9)	1 (2.3)	1 (10)	< 0.001
Active Disease		87/174 (50)	75/121 (62)	8 (18.6)	4 (40)	< 0.001

*IQR* Interquartile range, *PartC* Partial Coverage, *FullC* Total Coverage, *PRI* Private System

*NSAID* Nonsteroidal anti-inflammatory drugs, *DMARD* Disease-modifying antirheumatic, *bDMARD* Biological therapy

^a^: Includes arthritis and myositis

^b^: Includes neurological, hematological, pulmonary, gastrointestinal, cardiovascular, nephritis, serositis, adenomegaly, hepatomegaly, hepatitis, thyroiditis and uveitis manifestations

* Analyzed with Chi square test

** Analyzed with Kruskal-Wallis test

### Impact on caregivers

**Fig. 1:**
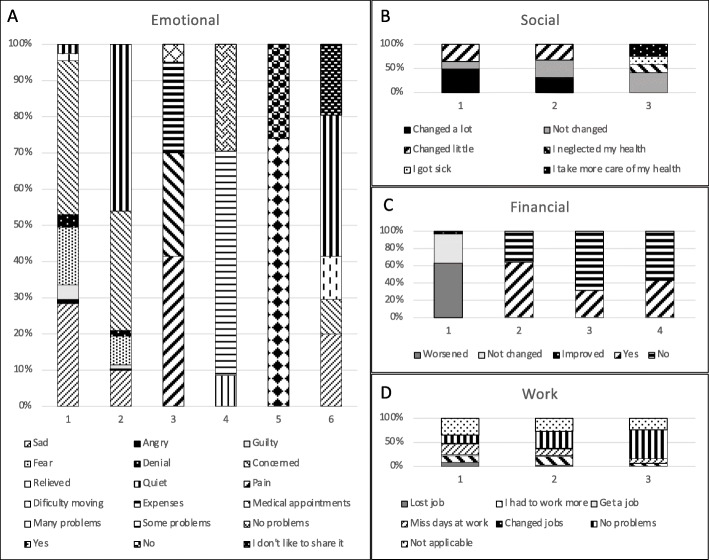
Emotional, Social, Financial and Work Impact of PRD on caregivers. Note: The numbers appearing on the x axis represent the items on each dimension. The captions show the participants’ answers. See Supplementary Table for reference

Caregivers took a median of ten (IQR 6–12) minutes to complete the CAREGIVERS questionnaire, and there was no data lost during collection. Detailed results and PRD comparisons are presented in Fig. 1, Supplementary Table and Supplementary Fig. 2.

#### I. Emotional Impact

Most of the caregivers felt concern (85, 42.5%) when learning about the diagnosis, which was then modified by tranquility (88, 44%) when the current feeling was questioned. However, 40 expressed sadness when sharing the patient’s PRD (20%) and 39 did not like to do so (19.5%).

The main cause of concern is pain (83, 41.5%), followed by difficulty in movement (57, 28.5%) and covering the costs of treatment (50, 25%). Furthermore, 141 thought about a future with problems in the patient’s life (70.5%), causing anguish (148, 74%).

#### II. Social impact

In 99 caregivers (49.5%), the use of their time changed a lot upon learning the PRD of the patient. In addition, 33 (16.5%) reported becoming ill and 36 (18%) have neglected their health since then. Social life varied according to the PRD. With JSLE, there was a significant change (25, 39.6%), but it did not change in JIA (48, 44%) and it slightly changed in JDM (15, 53.5%, *p <* 0.01).

#### IIIA. Financial impact

The family financial situation worsened upon diagnosis of the patient in most cases (JIA 63 [57.8%], JSLE 19 [69.8%] and JDM 44 [67.8%], *p =* 0.27). Almost two thirds had to borrow money, more frequently with JSLE (48 [76.1%] vs JIA 62 [56.8%] and JDM 19 [67.8%], *p =* 0.03); 63 stopped buying medicines due to lack of money (31.5%), and 86 received additional financial support for the treatment (43%).

#### IIIB. Work impact

Almost half of the caregivers had problems at work after the diagnosis of the patient (97, 48.5%), regardless of the PRD. The labor impact was lower in the partner and family (76 [38%] and 16 [8%], respectively).

#### IV. Family Impact

Family relationships changed in 81 caregivers (25 [12.5%] worsened and 56 [28%] improved). Most asked for help because of the PRD (127, 63.5%), with the partner or family being the main support network (60 [30%] and 65 [32%], respectively). Despite this, 118 caregivers went to medical appointments unaccompanied (59%).

#### V. Impact on the Caregiver-Patient Relationship

The study found that 142 (71%) caregivers improved the relationship with the patient, especially in the JSLE group (56 [88.9%] vs JIA 68 [62.4%] and JDM18 [64.3%], *p <* 0.01).

#### VI. Impact on the Relationship of the Partners

In 38.5% of the cases, relationships became closer as a couple after the diagnosis of the patient (77 caregivers), while 32 (16%) had problems (divorce, separation or withdrawal), with no difference between PRD.

#### VII. Impact on Spirituality / Religion / Personal beliefs

The study found that 82 participants (41%) became more spiritual, while only nine moved away or abandoned religious practices (4.5%), regardless of the PRD.

#### VIII. Impact of Social Networks

Most searched for information about PRD on the internet (163, 81.5%); the JDM group did it less frequently (16 [57.1%] vs those of JIA 91 [83.5%] and JSLE [56] 88.8%, *p =* 0.02), and found it less useful (*p =* 0.02). Fewer used social networks to communicate with other caregivers (JIA 14 [12.8%], JDM 2 [7.1%] and JSLE 22 [34.9%], *p <* 0.01), although it was useful for most of them (33/38, 86.8%).

### Impact by dimensions of the CAREGIVERS questionnaire and sociodemographic, clinical and healthcare system variables

The information presented below refers to the post-consensus analysis of the above described scoring (Table 5). An additional description can be found in Supplementary Results.

**Table 5:** Impact from dimensions of the CAREGIVERS questionnaire and sociodemographic, clinical and healthcare system variables

		Global	Emotional	Social	Financial	Work	Family	Patient#	Couple°	Spirituality	Networks&
Total *n* = 200		36 (31–41)	11 (10–13)	6 (4–7)	3 (2–4)	6 (1–7)	8 (7–10)	0 (0–1)	0 (0–1)	1 (0–1)	0 (0–4)
Caregiver’s gender (Male)*		37 (33–40)	11 (10–13)	5 (4–7)	3 (2–4)	7 (6–8)^a^	8 (6–9)	0 (0–1)	0 (0–1)	0 (0–1)	0 (0–4)
Patient’s gender (Male)*		37 (33–42)	12 (11–13)^a^	6 (4–7)	3 (2–4)	6 (0–7)	9 (7–10)	0 (0–1)	1 (0–1)	1 (0–1)	0 (0–4)
Housewife*		36 (29–40)^c^	11 (10–13)	5.5 (4–7)	3 (1–4)	1 (0–6)^b^	9 (8–11)^c^	0 (0–1)	0 (0–1)	1 (0–1)	0 (0–4)
Paid employment*		36 (32–42)^c^	11 (10–13)	6 (4–7)	3 (1–4)	6 (6–7)^b^	8 (6–9)^c^	0 (0–1)	0 (0–1)	1 (0–1)	0 (0–4)
Partner (No)*		39 (33–44)^b^	11 (10–13)	6.5 (5–7)^a^	4 (3–5)^a^	6 (6–7)	8.5 (6–11)	0 (0–1)	0 (0–0)^a^	1 (0–1)	0.5 (0–4)^a^
Education ≤9 years*		36 (31–40)	11 (10–13)	6 (4–7)	4 (2–4)^b^	6 (0–7)^a^	8 (8–10)	0 (0–0)	0 (0–1)	1 (0–1)	0 (0–3)^c^
Education > 9 years*		36 (32–42)	11 (10–13)	5 (4–7)	3 (1–4)^b^	7 (6–7)^a^	8 (6–10)	0 (0–1)	0 (0–1)	1 (0–1)	0 (0–4)^c^
JIA**		36 (31–41)	11 (10–13)	5 (4–7)^a^	3 (1–4)^a^	6 (1–7)	9 (7–10)	0 (0–1)^a^	1 (0–1)	1 (0–1)^c^	0 (0–4)
JDM**		33.5 (30–39)	11 (9–12)	5.5 (4–6)^a^	4 (2–4)^a^	6 (0–7)	6 (6–9)	0 (0–1)^a^	0 (0–1)	0.5 (0–1)^c^	0 (0–1)
JSLE**		36 (33–42)	11 (10–13)	6 (5–8)^a^	3 (3–4)^a^	6 (1–7)	8 (6–10)	0 (0–0)^a^	0 (0–1)	0 (0–1)^c^	0 (0–4)
Cutaneous features*		37.5 (32–42)	11 (10–13)	6 (5–7)^a^	4 (3–4)^a^	6 (0–7)	8.5 (7–10)	0 (0–0)^c^	0 (0–1)	0.5 (0–1)	0 (0–4)
Musculoskeletal features*^d^		36 (31–41)	11 (10–13)	5 (4–7)	3 (2–4)	6 (0–7)	9 (7–10)	0 (0–1)^a^	0 (0–1)	1 (0–1)	0 (0–4)
Systemic features*^e^		37 (32–42)	11.5 (10–13)	6 (5–7)^a^	4 (2–4)^a^	6 (1–7)	8.5 (7–11)	0 (0–0)^a^	0 (0–1)	1 (0–1)	0 (0–4)
Hospitalization (Yes)*		36 (31–40)	11 (10–13)	6 (4–7)^c^	3.5 (2–4)^a^	6 (0–7)	8 (6–10)	0 (0–0)^a^	0 (0–1)	1 (0–1)	0 (0–4)
Disability (Yes)*		36 (31–40)	11 (10–13)	6 (5–8)^a^	4 (3–5)^a^	6 (0–6)^a^	9 (8–10)	0 (0–1)	0 (0–1)	0 (0–1)	0 (0–4)
Active Disease (Yes)*		36 (32–41)	11 (10–13)	6 (5–7)^a^	4 (3–4)^a^	6 (0–6)^a^	9 (7–10)	0 (0–1)	0 (0–1)	1 (0–1)	0 (0–4)
Treatment (Yes)*	NSAID	37 (31–42)^c^	11 (10–13)^c^	6 (4–7)	3 (2–4)^a^	6 (0–7)^a^	9 (8–10)^c^	0 (0–1)	1 (0–1)^a^	1 (0–1)	0 (0–4)
	DMARD	36 (32–40)	11 (10–13)	5 (4–7)	3 (1–4)^c^	6 (1–7)	8 (7–10)	0 (0–1)	0 (0–1)	1 (0–1)	0 (0–4)
	GC	36.5 (32–41)	11 (10–13)	6 (5–7)^a^	4 (3–4)^b^	6 (0–7)	8 (7–10)	0 (0–0)^a^	0 (0–1)	0.5 (0–1)	0 (0–4)
	bDMARD (No)	36 (32–41)^a^	11 (10–13)	6 (4–7)^a^	3 (2–4)^a^	6 (0–7)	9 (7–11)^c^	0 (0–1)	0 (0–1)	1 (0–1)	0 (0–4)
PartC**		37 (32–41)^a^	11 (10–13)	6 (5–7)^b^	4 (3–4)^b^	6 (0–6)^b^	9 (8–10)^a^	0 (0–1)^a^	0 (0–1)	1 (0–1)	0 (0–4)
FullC**		33 (31–36)^a^	11 (10–12)	4 (3–6)^b^	1 (1–2)^b^	7 (6–7)^b^	8 (6–9)^a^	0 (0–1)^a^	0 (0–1)	1 (0–1)	0 (0–2)
PRI**		34.5 (28–45)^a^	12.5 (9–13)	4.5 (3–6)^b^	1.5 (1–3)^b^	7 (1–8)b	7.5 (6–10)^a^	1 (0–1)a	0.5 (0–1)	1 (0–1)	2 (0–4)
More than one hour to the center *		36 (32–40)	11 (10–13)	6 (4–7)^a^	3.5 (2–4)^a^	6 (0–7)^c^	9 (8–10)^a^	0 (0–1)	0 (0–1)	1 (0–1)	0 (0–2)^a^

Note: All values are expressed as median (IQR). *IQR* Interquartile range, *JIA* Juvenile Idiopathic Arthritis, *JSLE* Juvenile Systemic Lupus Erythematosus, *JDM* Juvenile Dermatomyositis, *NSAID* Nonsteroidal anti-inflammatory drugs, *DMARD* Disease-modifying antirheumatic drugs, *GC* Systemic Glucocorticoids, *bDMARD* Biological therapy

*PartC* Partial Coverage Healthcare system, *FullC* Total Coverage Healthcare system, *PRI* Private Healthcare System

^a^ *p <* 0.05

^b^ *p <* 0.001

^c^ Tendency

^d^ Includes arthritis and myositis

^e^ Includes neurological, hematological, pulmonary, gastrointestinal, cardiovascular, nephritis, serositis, adenomegaly, hepatomegaly, hepatitis, thyroiditis and uveitis manifestations

* Analyzed with Chi square test

** Analyzed with Kruskal-Wallis test

# Relationship of the caregiver with the patient

° Relationship of the caregiver with partner

& Social Network

Participants presented an intermediate global score in the questionnaire (36, IQR 31–41). A higher burden of PRD was found in those with PartC and less in those with a partner and in the caregivers of patients treated with bDMARD.

The emotional impact increased in caregivers of male patients (12 [IQR 11–13) vs 11 [10–13], *p =* 0.01). The social dimension showed significant differences regarding PRD, healthcare system, time to reach the center, presence of disability, active disease, cutaneous and systemic manifestations, use of GC or bDMARD and partner.

Differences were observed in all the variables assessed in the caregiver’s financial impact; only the presence of musculoskeletal manifestations, DMARD use, gender, and occupation showed no change. The labor impact was considerably greater in male caregiver with paid work and with higher education levels. The family impact increased in PartC and in those who need more time to get to the hospital, while the relationship with the patient had a higher PRI score.

No variables affecting spirituality were found. For caregivers without a partner, the impact of social networks increased.

## Discussion

The use of a validated questionnaire to measure multiple dimensions (such as the emotional, social, economic, family, partner and the patient-caregiver relationship) of the lives of caregivers allows for the documenting of the significant impacts of the PRD. The sample reflects the socioeconomic, geographic and healthcare diversity in Mexico. The levels of fragmentation in the health-care system represents the distribution in the country. The impact is greater in patients with a PartC, where the state covers only part of the costs, and, therefore, generates an out-of-pocket cost for the patient’s family. These findings have been previously documented in adults with rheumatic diseases [25]. The distribution of patients with JSLE and JDM is lower in PRI, and this is probably derived from the high costs that these diseases represent, as well as from the low proportion of health insurance coverage of major medical expenses in the Mexican population. Interestingly, a large proportion of patients were in remission in FullC, where the possibility of obtaining specialized treatments without out-of-pocket costs is present. In fact, the global impact (affecting 5 of 8 dimensions) was considerably greater in PartC than in the rest of the health-care systems, certainly related to more unfavorable social conditions.

These results confirm inequality in health-care access and coincide with those reported from a study of the difficulties that caregivers face in the Moroccan population [26], which had a smaller sample and was focused only on patients with JIA. In the case of the present study, this problem is evident in the three PRD included.

The impact of the diagnosis was high in all the caregivers, distributed similarly in the three PRD evaluated. However, there were slight differences in the social and financial impacts. Furthermore, these differences were associated with other clinical variables such as systemic manifestations, previous hospitalizations, disability or active disease. A greater economic impact was clear in patients with use of GC and bDMARD, although the emotional impact was less in those with bDMARD, which we assume is due to a better therapeutic response. Also, data showed an increase in social impact in caregivers of patients with cutaneous manifestations. It is assumed that this may be due to the medical recommendation to avoid sun exposure, especially in hot regions such as in the northeast, or to a secondary social stigmatization effect of the presence of visible physical features in patients. This confirms the significant impact of the diagnosis of a PRD on families and the need to carry out comprehensive interventions along with the standard treatment of patients.

The social and financial dimensions were the most affected by the different variables. Two of the main determinants of the impact of PRD on this population were PartC as health system and those without partners; both with unfavorable clinical and demographic situations. As well, caregivers of patients with active disease showed a higher impact on social, financial, and work domains.

Furthermore, living more than an hour away from the care center had an impact on the social, financial, work and family dimensions, as well as on the opportunity to access social networks to search for information. This is a result that has been previously documented in economic impact studies on rheumatic diseases in adults [27]. This finding will need to be further addressed in the pediatric population in different societies and health-care systems through a specific study in the future.

Another important finding is the difference in impact as a result of the gender of the caregiver. Researchers observed a greater economic, labor and financial impact on men, coinciding with the differences in roles in Mexican society, where men are providers. Regarding the patient, a greater emotional impact was observed in those whose children were boys. This could be explained by the greater severity of these diseases in males, although it is less marked in the pediatric population than in that of adults [28–32]. This could also be related to the different expectations that are held in the future for a woman or a man in Mexican society. Moreover, a higher percentage of female caregivers were found to be housewives. A third reported not having additional help, and this was linked to having less time to take care of their health and worsening of their social life (Supplementary Table). These findings reflect social inequities and should be explored further with a study designed from a gender perspective.

Results show that the family impact was less than expected, with only 12.5% reporting the worsening of the family relationship. Differences related to the presence of a partner were identified, and most were positive. In 65% of the participants, the relationship did not change or became closer, and only 17% reported separation as a result of the PRD. Similarly, 71% of caregivers reported an improvement in the relationship with the patient, especially in those with JSLE (Supplementary Table). These findings can be explained because families are generally very close in Mexico, and this benefits the approach to difficult situations, such as the diagnosis of a PRD.

Regarding the impacts on spirituality, greater changes were expected when the study was designed. It was thought that this dimension would have a significant differential impact because spirituality represents a social and cultural pillar in Mexico. However, results show that only 41% of caregivers turned to religion after the diagnosis of PRD. It would be interesting to evaluate this dimension in countries with a greater spiritual or religious diversity than Mexico.

Lastly, regarding the impact of social networks, results show that 81% of the participants looked for information about the PRD on social networks, but with an ambivalent relationship toward its use (27.5% generated anxiety and 30% helped). This finding is relevant because it is important to encourage caregivers to be informed in order to make better decisions. However, it is necessary to guide them in accessing information that will help them increase knowledge and avoid content that generates negative feelings about patient care.

The main limitation of this study is that the CAREGIVERS questionnaire is validated only for JIA. However, we considered that JSLE and JDM share substantial clinical features with JIA (e.g., pain, fatigue, and disability) that drive us to include them all in this study. Besides, the considerably smaller number of patients with JSLE and JDM in our country make it difficult for us to conduct both a validation and an observational study. Therefore, we decided to assume this bias in order to gain relevant information from a wide range of caregivers. We understand that some adjustments need to be made to the questionnaire before being used systematically in all PRD, but it can be useful to show some of the most relevant aspects impacted by these conditions on caregivers’ lives, as demonstrated by this report.

Selection bias is present as a result of the exclusion of caregivers of patients that went through hospitalizations in the last month prior to recruiting. This criterion was added to avoid recent extreme anxiety events which would have resulted in unrepresentative outcomes on the impact of the disease over time, which was the main objective of the CAREGIVERS questionnaire. Additionally, some important factors, such as age at diagnosis and duration of the disease, were missed. Despite this limitation, the results show higher scores in those with a history of hospitalizations. Therefore, the questionnaire could be useful to measure the impact at different times during the history of PRD, including critical events such as hospitalizations and flares.

Finally, another limitation is that a specific and validated tool was not used to define disability or disease activity. Instead, the judgment of the treating physician that was reported in the clinical record or the perception of the caregiver was considered. This decision was made in order to avoid overwhelming the participants with more questionnaires, but it is necessary to consider including more precise measurements of these aspects in future works derived from this study.

## Conclusion

This study highlights the need of support for caregivers of children with PRD, showing that the influence of sociodemographic factors can be devastating on those families. Although all the participants are Mexican, findings can be generalized to caregivers of patients with PRD and similar sociodemographic factors in other regions, especially Latin America, Africa and Asia. Decisively, these data will help physicians to identify the areas with the greatest need for intervention in order to achieve comprehensive care for caregivers and their patients.

## Supplementary Information

**Additional file 1: Supplementary Fig. 1**. Geographic distribution of the participants. **Supplementary Fig. 2**. Family, Couple, Patient, Spirituality and Social Networks Impact of PRD on caregivers. **Supplementary Table**. Results of the CAREGIVER questionnaire. Impact of PRD on caregivers compared by disease. **Supplementary Results**. Complementary description of the impacts by dimensions of the CAREGIVERS questionnaire.

## Data Availability

The datasets used and/or analyzed during the current study are available from the corresponding author on reasonable request.

## Abbreviations

PRD: Pediatric rheumatic diseases
JIA: Juvenile Idiopathic Arthritis
JDM: Juvenile Dermatomyositis.
JSLE: Juvenile Systemic Lupus Erythematosus
CAREGIVERS questionnaire: Impact of Pediatric Rheumatic Diseases on Caregivers Multi-assessment Questionnaire
PartC: Partial coverage
FullC: Full coverage
PRI: Private coverage
NSAID: Non-steroidal anti-inflammatory drugs
DMARD: Disease modifying drugs and synthetic immunosuppressants
GC: Systemic glucocorticoids
bDMARD: Biological immunosuppressive therapies
IQR: Interquartile range
