# Supplementary material for: Psychosocial and economic impact of rheumatic diseases on caregivers of Mexican children
Source: Pediatr Rheumatol Online J. 2021 Mar 17;19:30. doi: 10.1186/s12969-021-00524-2 (PMC7967951; doi:10.1186/s12969-021-00524-2)
Supplement: Supplementary file 1 — Additional file 1: Supplementary Fig. 1. Geographic distribution of the participants. Supplementary Fig. 2. Family, Couple, Patient, Spirituality and Social Networks Impact of PRD on caregivers. Supplementary Table. Results of the CAREGIVER questionnaire. Impact of PRD on caregivers compared by disease. Supplementary Results. Complementary description of the impacts by dimensions of the CAREGIVERS questionnaire. [file 12969_2021_524_MOESM1_ESM.docx]

**Supplementary Figure 1. Geographic distribution of the participants.**


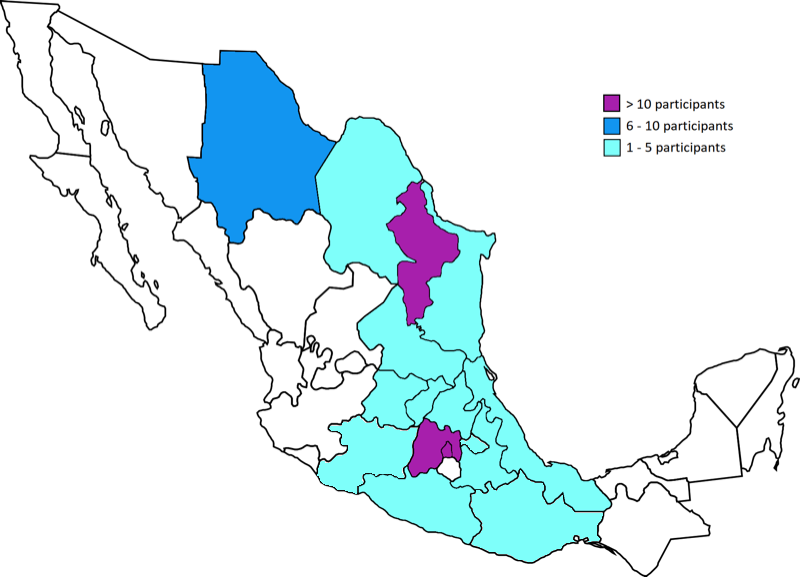


**Supplementary Figure 2. Family, Couple, Patient, Spirituality and Social Networks Impact of PRD on caregivers.**

**
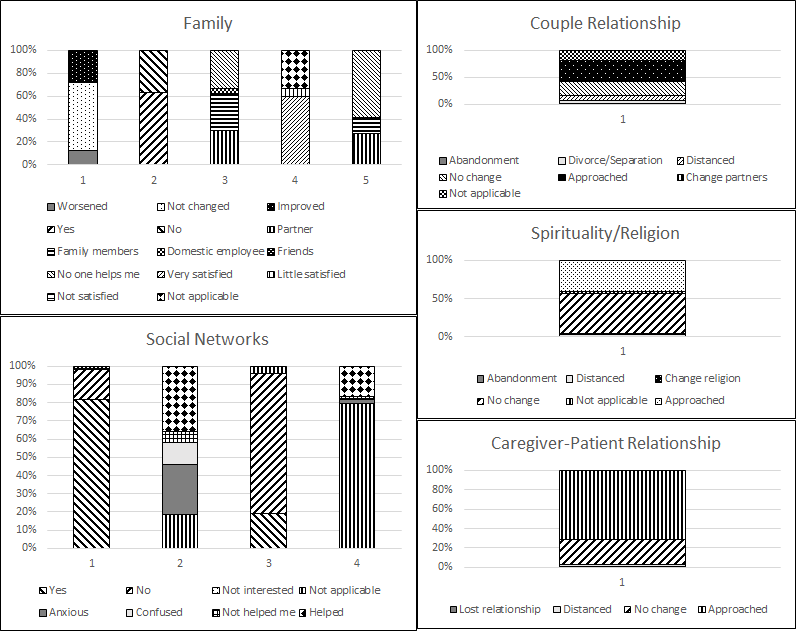
**

Note: The numbers appearing on the x axis represent the items on each dimension. The captions show the participants’ answers. See Supplementary Table for reference.

**Supplementary Table. Results of the CAREGIVER questionnaire. Impact of PRD on caregivers compared by disease.**

| Dimension | Total  n=200 | JIA  n=109 | JDM  n=28 | JSLE  n=63 | P Value |
| --- | --- | --- | --- | --- | --- |
| **I. Emotional Impact** |  |  |  |  |  |
| **1. How did you feel when you learned that your child/patient had a rheumatic disease?** |  |  |  |  | 0.62 |
| 1. Sad (4 points) | 57 (28.5) | 36 (33) | 7 (25) | 14 (22.2) |  |
| 2. Angry (3 points) | 2 (1) | 0 | 1 (3.6) | 1 (1.6) |  |
| 3. Guilty (3 points) | 8 (4) | 5 (4.5) | 1 (3.6) | 2 (3.1) |  |
| 4. Afraid (3 points) | 32 (16) | 15 (13.8) | 4 (14.3) | 13 (20.6) |  |
| 5. I did not accept this reality.(Denial) (5 points) | 7 (3.5) | 3 (2.8) | 0 | 4 (6.3) |  |
| 6. Concerned (3 points) | 85 (42.5) | 44 (40.4) | 15 (53.6) | 26 (41.2) |  |
| 7. It did not affect me. (1 point) | 0 | 0 | 0 | 0 |  |
| 8. Relieved (2 points) | 4 (2) | 3 (2.8) | 0 | 1 (1.6) |  |
| 9. Calmed (2 points) | 5 (2.5) | 3 (2.8) | 0 | 2 (3.1) |  |
| **2. At this time, how do you feel about your child/patient having a rheumatic disease?** |  |  |  |  | 0.88 |
| 1. Sad (4 points) | 19 (9.5) | 12 (11) | 2 (7.1) | 5 (8) |  |
| 2. Angry (3 points) | 1 (0.5) | 0 | 0 | 1 (2) |  |
| 3. Guilty (3 points) | 2 (1) | 1 (0.9) | 0 | 1 (2) |  |
| 4. Afraid (3 points) | 15 (7.5) | 8 (7.3) | 2 (7.1) | 5 (8) |  |
| 5. I do not accept this reality. (Denial) (5 points) | 3 (1.5) | 1 (0.9) | 0 | 2 (3.1) |  |
| 6. Concerned (3 points) | 63 (31.5) | 34 (31.1) | 10 (35.7) | 19 (30.1) |  |
| 7. It did not affect me. (1 point) | 0 | 0 | 0 | 0 |  |
| 8. Relieved (2 points) | 0 | 7 (6.4) | 0 | 2 (3.1) |  |
| 9. Calmed (2 points) | 88 (44) | 46 (42.2) | 14 (50) | 28 (44.4) |  |
| **3. What worries you the most about your child's/patient’s rheumatic disease?** | Contextual |  |  |  | 0.38 |
| 1. That he/she feels pain (0 points) | 83 (41.5) | 42 (38.5) | 11 (39.2) | 30 (47.6) |  |
| 2. That he/she has difficulty moving (running, walking) (0 points) | 57 (28.5) | 37 (34) | 8 (28.5) | 12 (19) |  |
| 3. Covering the expenses of the treatment (0 points) | 50 (25) | 23 (21.1) | 8 (28.5) | 19 (30.1) |  |
| 4. Being able to attend medical appointments (0 points) | 10 (5) | 7 (6.4) | 1 (3.5) | 2 (3.1) |  |
| **4. What do you think the future of your child/patient will be with a rheumatic disease?** |  |  |  |  | 0.38 |
| 1. With many problems in his/her life (3 points) | 17 (8.5) | 7 (6.4) | 2 (7.1) | 8 (12.7) |  |
| 2. With some problems in his/her life (2 points) | 124 (62) | 73 (66.9) | 15 (53.5) | 36 (57.1) |  |
| 3. No problems in his/her life (1 points) | 59 (29.5) | 29 (26.6) | 11 (39.2) | 19 (30.1) |  |
| **5. Does this cause you anxiety or stress?** (Yes) (1 point) | 148 (74) | 82 (75.2) | 20 (71.4) | 17 (27) | 0.88 |
| **6. How do you feel about sharing that your child/patient has a rheumatic disease with other people?** |  |  |  |  | 0.98 |
| 1. I feel sad. (4 points) | 40 (20) | 21 (19.2) | 5 (17.8) | 14 (22.2) |  |
| 2. I feel ashamed. (5 points) | 0 | 0 | 0 | 0 |  |
| 3. I worry that they make fun of him/her. (3 points) | 19 (9.5) | 10 (9.1) | 2 (7.1) | 7 (11.1) |  |
| 4. I don't like to share it. (4 points) | 39 (19.5) | 23 (21) | 6 (21.4) | 10 (15.8) |  |
| 5. I feel relieved. (2 points) | 24 (12) | 11 (10) | 4 (14.3) | 9 (14.2) |  |
| 6. I feel calm. (2 points) | 78 (39) | 44 (40.3) | 11 (39.2) | 23 (36.5) |  |
| **II. SOCIAL IMPACT** |  |  |  |  |  |
| **1. Have you changed the way you spend your time since you learned about your child's/patient’s rheumatic disease?** |  |  |  |  | 0.26 |
| 1. It has changed a lot. (3 points) | 99 (49.5) | 47 (43.1) | 14 (50) | 38 (60.3) |  |
| 2. It has not changed. (1 points) | 30 (15) | 17 (15.6) | 5 (17.8) | 8 (12.7) |  |
| 3. It has changed little. (2 points) | 71 (35.5) | 45 (41.2) | 9 (32.1) | 17 (27) |  |
| **2. Has your social life changed (walking, traveling, friends, get togethers, parties, etc.) since you learned about your child's/patient’s rheumatic disease?** |  |  |  |  | < 0.01 |
| 1. It has changed a lot. (3 points) | 64 (32) | 33 (30.2) | 6 (21.4) | 25 (39.6) |  |
| 2. It has not changed. (1 point) | 70 (35) | 48 (44) | 7 (25) | 15 (23.8) |  |
| 3. It has changed little. (2 points) | 66 (33) | 28 (25.7) | 15 (53.5) | 23 (36.5) |  |
| **3. Has your health changed since you learned about your child's/patient’s rheumatic disease?** |  |  |  |  | 0.12 |
| 1. I neglected my health. (2 points) | 36 (18) | 13 (11.9) | 6 (21.4) | 17 (27) |  |
| 2. I got sick. (3 points) | 33 (16.5) | 19 (17.4) | 2 (7.1) | 12 (19) |  |
| 3. It did not change. (1 point) | 82 (41) | 51 (46.8) | 12 (42.8) | 19 (30.1) |  |
| 4. I take more care of my health. (0 points) | 49 (24.5) | 26 (23.8) | 8 (28.5) | 15 (23.8) |  |
| **III. ECONOMIC IMPACT** |  |  |  |  |  |
| **1. Has your family’s economic situation changed since you learned about your child's/patient’s rheumatic disease?** |  |  |  |  | 0.27 |
| 1. The economic situation worsened. (2 points) | 126 (63) | 63 (57.8) | 19 (67.8) | 44 (69.8) |  |
| 2. The economic situation did not change. (1 point) | 68 (34) | 44 (40.3) | 8 (28.5) | 16 (25.4) |  |
| 3. The economic situation improved. (0 points) | 6 (3) | 2 (1.8) | 1 (3.5) | 3 (4.7) |  |
| **2. Have you, your partner or someone in your family had to borrow money to treat your child's/patient’s rheumatic disease?**  (Yes) (1 point) | 129 (64.5) | 62 (56.8) | 19 (67.8) | 48 (76.1) | 0.03 |
| **3. Have you stopped buying your child's/patient's medications because of lack of money?**  (Yes) (1 point) | 63 (31.5) | 28 (25.6) | 12 (42.8) | 23 (36.5) | 0.12 |
| **4. Have you received additional support (money, medications, food, etc.) to treat your child's/patient’s rheumatic disease?**  (Yes) (1 point) | 86 (43) | 41 (37.6) | 12 (42.8) | 33 (52.3) | 0.16 |
| **III.B LABOR IMPACT** |  |  |  |  |  |
| **1. Have you had problems at work because of attending to your child's/patient’s rheumatic disease?** |  |  |  |  | 0.66 |
| 1. I lost my job. (5 points) | 16 (8) | 9 (9.2) | 2 (7.1) | 5 (8) |  |
| 2. I had to work more. (4 points) | 27 (13.5) | 12 (11.0) | 6 (21.4) | 9 (14.3) |  |
| 3. I had to get a job. (4 points) | 6 (3) | 3 (2.7) | 1 (3.5) | 2 (3.1) |  |
| 4. I had to miss days at work. (2 points) | 44 (22) | 26 (23.8) | 3 (10.7) | 15 (23.8) |  |
| 5. I have changed jobs. (3 points) | 4 (2) | 0 | 1 (3.5) | 3 (4.7) |  |
| 6. I have had no problems at work. (1 point) | 33 (16.5) | 19 (17.4) | 4 (14.3) | 10 (15.8) |  |
| 7. Does not apply (0 points) | 70 (35) | 40 (36.7) | 11 (39.2) | 19 (30.1) |  |
| **2. Has your partner had problems at work because of attending to your child's/patient’s rheumatic disease?** |  |  |  |  | 0.16 |
| 1. He/she has lost his/her job. (5 points) | 6 (3) | 3 (2.7) | 0 | 3 (4.7) |  |
| 2. He/she has had to work more. (4 points) | 35 (17.5) | 24 (22) | 2 (17.8) | 6 (9.5) |  |
| 3. He/she had to get a job. (4 points) | 5 (2.5) | 3 (2.7) | 1 (3.5) | 1 (2.0) |  |
| 4. You have had to miss your job. (2 points) | 26 (13) | 10 (9.1) | 5 (17.8) | 11 (17.4) |  |
| 5. He/she has changed jobs. (3 points) | 4 (2) | 2 (1.8) | 1 (3.5) | 1 (2) |  |
| 6. He/she has had no problems at work. (1 point) | 69 (34.5) | 45 (41.2) | 6 (21.4) | 18 (28.5) |  |
| 7. Does not apply (0 points) | 55 (27.5) | 22 (20.1) | 10 (35.7) | 23 (36.5) |  |
| **3. Has anyone in your family had problems at work because of attending to your child’s/patient’s rheumatic disease?** |  |  |  |  | 0.16 |
| 1. Someone has lost his/her job. (5 points) | 0 | 0 | 0 | 0 |  |
| 2. Someone has had to work more. (4 points) | 6 (3) | 2 (1.8) | 3 (10.7) | 1 (2) |  |
| 3. Someone has had to get a job. (4 points) | 1 (0.5) | 1 (0.9) | 1 (3.5) | 0 |  |
| 4. Someone has had to miss work. (2 points) | 9 (4.5) | 4 (3.6) | 5 (17.8) | 4 (6.3) |  |
| 5. Someone has had to changed jobs. (3 points) | 0 | 0 | 0 | 0 |  |
| 6. No one has had problems at work. (1 point) | 60 (30) | 39 (35.7) | 0 | 6 (25.4) |  |
| 7. Does not apply (0 points) | 24 (62) | 63 (57.8) | 19 (67.8) | 42 (66.6) |  |
| **IV. FAMILY IMPACT** |  |  |  |  |  |
| **1. Has the way in which you interact with your family changed since you learned about your child's/patient’s rheumatic disease?** |  |  |  |  | 0.18 |
| 1. It has worsened. (2 points) | 25 (12.5) | 17 (15.6) | 2 (7.1) | 6 (9.5) |  |
| 2. It has not changed. (1 point) | 119 (59.5) | 64 (58.7) | 21 (75) | 34 (53.9) |  |
| 3. It has improved. (0 points) | 56 (28) | 28 (25.7) | 5 (17.8) | 23 (36.5) |  |
| **2. Do you need, or have you needed to ask your family for help in order to attend to the rheumatic disease of your child/patient?**  (Yes) (1 point) | 127 (63.5) | 68 (62.4) | 16 (57.1) | 43 (68.2) | 0.56 |
| **3. During the rheumatic illness of your child/patient, who helps you with domestic activities?** |  |  |  |  | 0.14 |
| 1. Partner (2 points) | 60 (30) | 32 (29.3) | 8 (28.5) | 20 (31.7) |  |
| 2. Family members (2 points) | 65 (32.5) | 28 (25.7) | 11 (39.2) | 26 (41.5) |  |
| 3. Housewife (4 points) | 8 (4) | 7 (6.4) | 1 (3.5) | 0 |  |
| 4. Friends (3 points) | 0 | 0 | 0 | 0 |  |
| 5. No one helps me. (5 points) | 67 (33.5) | 42 (38.5) | 8 (28.5) | 17 (26.9) |  |
| **4. How satisfied are you with this help?** |  |  |  |  | 0.23 |
| 1. Very satisfied (0 points) | 120 (60) | 62 (56.8) | 19 (67.8) | 39 (61.9) |  |
| 2. Little satisfied (2 points) | 13 (6.5) | 5 (5) | 1 (3.5) | 7 (11.1) |  |
| 3. Not satisfied (3 points) | 1 (0.5) | 0 | 0 | 1 (2.0) |  |
| 4. Does not apply (0 points) | 66 (33) | 42 (38.5) | 8 (28.5) | 16 (25.4) |  |
| **5. Who accompanies you to your child's/patient’s medical appointments?** |  |  |  |  | 0.97 |
| 1. Family members (2 points) | 26 (13) | 13 (11.9) | 4 (14.3) | 9 (14.3) |  |
| 2. Partner (2 points) | 55 (27.5) | 31 (28.4) | 8 (28.5) | 16 (25.4) |  |
| 3. Housewife (4 points) | 0 | 0 | 0 | 0 |  |
| 4. Friends (3 points) | 1 (0.5) | 1 (0.9) | 0 | 0 |  |
| 5. No one accompanies me. (5 points) | 118 (59) | 64 (58.7) | 16 (57.1) | 38 (60.3) |  |
| **V. IMPACT ON CAREGIVER-PATIENT RELATIONSHIP** |  |  |  |  |  |
| **1.Has the relationship with your child/patient changed since the diagnosis of his/her rheumatic disease?** |  |  |  |  | < 0.01 |
| 1. We have lost the relationship. (3 points) | 2 (1) | 2 (1.8) | 0 | 0 |  |
| 2. We have moved away. (2 points) | 4 (2) | 1 (0.9) | 1 (3.5) | 2 (3.1) |  |
| 3. Our relationship has not changed. (1 points) | 52 (26) | 38 (34.8) | 9 (32.1) | 5 (8) |  |
| 4. We are closer. (0 points) | 142 (71) | 68 (62.4) | 18 (64.3) | 56 (88.9) |  |
| **VI. PARTNER RELATIONSHIP IMPACTS** |  |  |  |  |  |
| **1. Has your relationship changed as a result of the rheumatic illness of your child/patient?** |  |  |  |  | 0.24 |
| 1. Abandonment (5 points) | 4 (2) | 2 (1.8) | 0 | 2 (3.1) |  |
| 2. Divorce/Separation (4 points) | 8 (4) | 3 (2.7) | 1 (3.5) | 4 (6.3) |  |
| 3. Distance (We distance ourselves as a couple.) (2 points) | 20 (10) | 13 (11.9) | 2 (7.1) | 5 (7.9) |  |
| 4. It has not changed. (1 point) | 53 (26.5) | 36 (33) | 9 (32.1) | 8 (12.7) |  |
| 5. We are closer than before. (0 points) | 77 (38.5) | 39 (35.7) | 11 (39.2) | 27 (42.8) |  |
| 6. Changed partners (3 points) | 1 (0.5) | 1 (0.9) | 0 | 0 |  |
| 7. I have no partner. (0 points) | 37 (18.5) | 15 (13.7) | 5 (17.8) | 17 (26.9) |  |
| **VII. IMPACT ON SPIRITUALITY / RELIGION / PERSONAL BELIEFS** |  |  |  |  |  |
| **1. Have your spirituality, religion or personal beliefs changed since the diagnosis of rheumatic disease of your child/patient?** |  |  |  |  | 0.16 |
| 1. I abandoned my religion/beliefs. (5 points) | 1 (0.5) | 0 | 0 | 1 (1.6) |  |
| 2. I feel further away from my religion/beliefs. (4 points) | 4 (2) | 2 (1.8) | 0 | 2 (3.2) |  |
| 3. I changed my religion/beliefs. (3 points) | 4 (2) | 2 (1.8) | 0 | 2 (3.2) |  |
| 4. I did not change religion/beliefs. (1 point) | 104 (52) | 67 (61.5) | 14 (50) | 23 (36.5) |  |
| 5. I have never believed in anything. (0 points) | 5 (2.5) | 3 (2.8) | 1 (3.6) | 1 (1.6) |  |
| 6. I am closer to religion and my beliefs. (0 points) | 82 (41) | 35 (32.1) | 13 (46.4) | 34 (54) |  |
| **VIII. IMPACT OF SOCIAL NETWORKS** |  |  |  |  |  |
| **1. Have you searched for information about your child's/patient’s rheumatic disease on the internet?** | Contextual. |  |  |  | 0.02 |
| 1. Yes (0 points) | 163 (81.5) | 91 (83.5) | 16 (57.1) | 56 (88.8) |  |
| 2. No (0 points) | 33 (16.5) | 15 (13.7) | 11 (39.2) | 7 (11.1) |  |
| 3. I am not interested. (0 points) | 1 (0.5) | 1 (0.9) | 0 | 0 |  |
| 4. Not applicable (I don't have internet.) (0 points) | 3 (1.5) | 2 (1.8) | 1 (3.5) | 0 |  |
| **2. How has the information about your child’s/patient’s rheumatic disease on the internet affected you?** |  |  |  |  | 0.02 |
| 1. It has caused me anxiety/stress. (4 points) | 55 (27.5) | 30 (27.5) | 6 (21.4) | 19 (30.1) |  |
| 2. It has confused me. (3 points) | 24 (12) | 11 (10) | 5 (17.8) | 8 (12.7) |  |
| 3. It has not helped me. (2 points) | 12 (6) | 8 (7.3) | 1 (3.5) | 3 (4.7) |  |
| 4. It has helped me. (0 points) | 72 (36) | 42 (38.5) | 4 (14.3) | 26 (41.2) |  |
| 5. Does not apply (0 points) | 37 (18.5) | 18 (16.5) | 12 (42.8) | 7 (11.1) |  |
| **3. Have you used social media to communicate with other parents/caregivers of children suffering from the same rheumatic disease as your child/patient?** | Contextual. |  |  |  | < 0.01 |
| 1. Yes (0 points) | 38 (19) | 14 (12.8) | 2 (7.1) | 22 (34.9) |  |
| 2. No (0 points) | 154 (77) | 91 (83.5) | 24 (85.7) | 39 (61.9) |  |
| 3. Does not apply (0 points) | 8 (4) | 4 (3.6) | 2 (7.1) | 2 (3.1) |  |
| **4. How did you feel about communicating with other people through social networks?** |  |  |  |  | 0.04 |
| 1. It caused me anxiety/stress. (4 points) | 4 (2) | 1 (0.9) | 0 | 1 (4.7) |  |
| 2. It confused me. (3 points) | 1 (0.5) | 1 (0.9) | 0 | 0 |  |
| 3. It has not helped me. (2 points) | 3 (1.5) | 2 (1.8) | 0 | 1 (1.6) |  |
| 4. It has helped me. (0 points) | 33 (16.5) | 13 (11.9) | 2 (7.1) | 18 (28.5) |  |
| 5. Does not apply (0 points) | 159 (79.5) | 92 (84.4) | 26 (92.8) | 41 (65) |  |

Notes: All data are presented as an absolute frequency and percentage in parentheses. There were no missing data during the application and collection of the questionnaires.

JIA: Juvenile Idiopathic Arthritis. JDM: Juvenile Dermatomyositis. JSLE: Juvenile Systemic Lupus Erythematosus. PRD: Pediatric Rheumatic Diseases. CAREGIVERS Questionnaire: Impact of Pediatric Rheumatic Diseases on Caregivers Multi-assessment Questionnaire.

**Supplementary Results. Complementary description of the impacts by dimensions of the CAREGIVERS questionnaire.**

The following summary describes the findings of the univariate analysis of the patients and the responses of the CAREGIVERS questionnaire prior to scoring by the researchers.

Results show that the feelings of emotion at diagnosis did not show differences with respect to the demographic variables of the caregivers, clinical manifestations, treatment, complications or type of health system. Upon applying the questionnaire, caregivers of patients with neurological manifestations felt more fear (20% vs. 7%, *p =* 0.02), while those with cardiovascular patients felt more guilt (14% vs. 0%, *p <* 0.01) when compared to those who did not present differences. Those caregivers of patients with active disease felt more concern (41% vs. 18%, *p =* 0.04).

Differences were found in the causes of concern in caregivers regarding the health care system. Additionally, caregivers felt more concerned with covering the costs of treatment with patients with skin manifestations or disabilities (32% vs. 19%, *p =* 0.01 and 42% vs. 19%, *p =* 0.02, respectively) and with joint conditions due to the difficulty the patients have with movement. (37 vs 8%, *p <* 0.001). Caregivers of patients with DMARD were concerned about pain (42% vs. 27%) and covering expenses (27% vs. 0%, *p =* 0.02), while those with biologic use had no concern. Fewer caregivers were concerned about being able to keep showing up to appointments, despite the fact that a large proportion required more than 1 hour to reach the center.

Caregivers of male patients predicted a future with more problems, and this generated more anxiety for these caregivers than for those who cared for female patients (82% vs. 64%, *p =* 0.02 and 93% vs. 65%, *p <* 0.001, respectively). There were no differences in this perception regarding the health care system, clinical manifestations, treatment, complications or disease activity of the patients, nor in relation to the sociodemographic variables of the caregivers.

Those caregivers that attended PartC suffered a greater impact on their use of time (65% vs. 28%, 33% and 20%, *p <* 0.001) and had no partner (64% vs. 42%, *p =* 0.04). A greater impact was seen in those who had patients with cardiovascular manifestations (79% vs. 46%, *p =* 0.04) that used corticosteroids (57% vs. 36%, *p =* 0.02) or were disabled (63% vs. 41%, *p =* 0.04). The use of biological therapy did not affect the use of their time (32% vs. 52%, *p =* 0.03). Caregivers that had to travel a greater distance to the center suffered a greater impact on their use of time (*p =* 0.03).

Results show that the social life of the participants changed mainly in those who attended PartC and PRI (78% and 70% vs 44% and 45%, *p <* 0.001) and had no partner (82% vs 57 %, *p =* 0.01). This was also true in caregivers of patients with skin manifestations (75% vs. 55%, *p =* 0.02), use of corticosteroids (78% vs. 50%, *p <* 0.001), were hospitalized (70% vs.% 2%, *p =* 0.04), had disability (74% vs. 58%, *p =* 0.04) and active disease at the time of applying the questionnaire (74% vs. 50%, *p =* 0.01).

Family finances worsened more frequently in caregivers of patients with corticosteroid use (71% vs. 53%, *p =* 0.02) and with an active disease (69% vs. 53%, *p =* 0.02). Furthermore, finances also worsened in caregivers with lower educational levels (basic education 69% vs. high school or higher 54%, *p =* 0.02). Caregivers required borrowing more frequently when their patients had cutaneous (76% vs. 54%, *p =* 0.01) or cardiovascular (93% vs. 61%, *p =* 0.01) manifestations, when they used corticosteroids (72% vs. 54%, *p =* 0.01), had been hospitalized (69% vs. 53%, *p =* 0.02), had disability (79% vs. 57%, *p =* 0.01) or an active disease (75% vs. 49%, *p <* 0.001). Moreover, caregivers without a partner (82% vs. 58%, *p =* 0.01), those who lived further from the center (*p =* 0.01) and those with less education (basic education 69% vs. high school or higher 56%, *p =* 0.01) borrowed money more frequently. Those with biological therapy use reported less borrowing (47% vs. 68%, *p =* 0.01). The same variables involved in the difficulty of purchasing medications and the need for additional support were obtained (this data is not shown).

Results show that male caregivers had to work more and missed work more often compared to women (33% vs. 11% and 30% vs. 21%, respectively, *p =* 0.01). Also, caregivers of patients with disabilities worked more (35% vs. 11%, *p =* 0.01).

Caregivers of patients with an active disease ask for more help from family members than those in remission (68% vs. 54%, *p =* 0.04). The same was true for caregivers with paid work (81% vs. 37%, *p =* 0.01), those who do not have a partner (81% vs. 19%, *p =* 0.01) and those who had more education (*p =* 0.03).

The relationship with the patient worsened more frequently when the patient was male (9% vs. 1%, *p =* 0.03) and improved mostly in those who were hospitalized (79% vs. 58%, *p =* 0.01). No variable influenced the relationship of the caregiver.

Caregivers were closer to religion when patients used DMARD (42% vs. 0%, *p <* 0.001) or corticosteroids (49% vs. 32%, *p =* 0.02), in addition to those who had hospitalization (45% vs. 31%, *p =* 0.04). Caregivers without a partner moved away from religion more frequently (11% vs. 2%, *p =* 0.01).

Researchers found that caregivers search for information on the Internet more if they have a higher level of education (schooling < 9 years 74% vs. ≥ 9 years 88%, *p =* 0.03). In this population, it was reported to be more useful (31% vs. 45%, *p =* 0.03). There were no differences in the use of social networks regarding the variables that were studied.
